# Editorial: Physiology and pathology of neuroglia

**DOI:** 10.3389/fncel.2023.1246885

**Published:** 2023-07-18

**Authors:** Daniel Reyes-Haro, Alejandro López-Juárez, Adrián Rodríguez-Contreras

**Affiliations:** ^1^Universidad Nacional Autónoma de México, Instituto de Neurobiología - UNAM, Campus Juriquilla, Juriquilla, QRO, Mexico; ^2^Department of Health and Biomedical Sciences, The University of Texas Rio Grande Valley, Brownsville, TX, United States; ^3^The Roxelyn and Richard Pepper Department of Communication Sciences and Disorders, School of Communication, Northwestern University, Evanston, IL, United States

**Keywords:** anorexia, Down syndrome, diabetes, Fetal Alcohol Syndrome, pain

Neuroglia is the largest population of cells in the brain and participates in formation, maintenance, and modulation of synaptic circuits. This heterogenous group includes macroglia (astroglia and oligodendroglia) and microglia. Neurons and neuroglia form assemblies that potentiate the cognitive capability of the brain. In this topic, nine articles highlight structural and functional roles of neuroglia in brain physiology, which is fundamental to better understand the biology of neurodevelopmental and neurodegenerative diseases.

Our understanding of brain connectivity evolves rapidly and the cytoarchitecture of underexplored brain regions such as the cerebellum need to be revisited (De Zeeuw et al., 2021). Hence, Gómez-González et al. reviewed the organization of a peculiar cerebellar glial niche located at the subventricular zone of the fourth ventricle. In addition to neurons and ependymal cells, this region is rich in microglia as well as macroglia including astrocytes, Bergmann glia, and oligodendrocyte lineage cells. Further transcriptional and functional heterogeneity within these glial populations is discussed. Despite scarce to non-proliferative activity, this region shares similarity with adult neurogenic niches throughout the brain and various stimulating questions remain to be explored. Furthermore, glial organization is adapted to this highly vascularized niche and contribute to the glioneurovascular unit, a structural and functional element where glial cells respond to stimulation by coupling to increased sensory activity through development (Biesecker et al., 2016; Koehler, 2021). In a perspective contributed by Konecny et al., they hypothesize that augmented neuronal activity is associated with angiogenic factor production and creates an environment of intermittent hypoxia, promoting the expression of hypoxia-inducible transcription factors (HIFs) by the glioneurovascular unit. However, mechanisms triggering glioneurovascular coupling during early sensory neurodevelopment are unknown; therefore, further research using non-invasive approaches is proposed.

Neuron-glia coupling is physiologically tied to volume regulation. Ionic gradients permit neurons to communicate electrically, and glial cells help them to regulate volume. Astrocytes express a variety of cotransport systems and ion channels to maintain brain homeostasis through mobilization of osmolytes (Walch and Fiacco, 2022). Ochoa-de la Paz and Gulias-Cañizo identify glial cells as master regulators of the tripartite synapse volume, a property that gives them an important role in maintaining homeostasis. Dysfunction of volume regulation leads to pathology in conditions such as edema, uremia, and diabetes, in which solute imbalances occur.

Neuron-glia communication is mediated by neurotransmitters including glutamate, the main excitatory neurotransmitter of the brain. This signaling occurs through ionotropic and metabotropic receptors expressed by neurons and glial cells. Particularly, astrocytes modulate neuronal activity through release of gliotransmitters like glutamate and D-serine (Reyes-Haro et al., 2010). Additionally, astrocytes express glutamate transporters to regulate the glutamatergic tone at the synaptic cleft and supply glutamine to neurons that convert it into glutamate or GABA to refill synaptic vesicles (Martínez-Lozada and Ortega, 2023). Dysfunction of the glutamate-glutamine shuttle results in excitotoxicity that has been linked to Alzheimer's and Huntington's diseases. Thus, Cuellar-Santoyo et al. summarize the astrocyte's contribution to glutamatergic neurotransmission in physiological and pathological conditions.

Astrocytes also respond to neuronal activity with calcium transients, a signaling mechanism that seems involved in pain and nociception (Prokhorenko and Smyth, 2023). Here, Higinio-Rodríguez et al. present an experimentally supported perspective in which coherent activity of astrocytes in pain-related brain areas plays critical roles in binding sensory, affective, and cognitive information, on a slow time scale. As astrocytes respond to noxious stimuli via calcium modulation likely independent of neuronal activation, this could represent the mechanism by which pain is created from nociception with the participation of astrocytes.

Glial cell responses in pathology involve inflammasomes, multi-protein intracellular signaling complexes which orchestrate inflammatory responses to a diverse range of pathogens and host-derived signals (Jewell et al., 2022). In their review article, Mata-Martínez et al., discuss aspects of the inflammatory process, focusing on accumulating evidence of multiprotein complexes that sense and respond in the context of inflammation. The authors argue that acute and chronic inflammation will engage a coordinated molecular response in various organs, involving glial cells in the brain. Interestingly, Down syndrome (DS) and Alzheimer's disease (AD) are characterized by chronic neuroinflammation, peripheral inflammation, astrogliosis, imbalanced excitatory/inhibitory neuronal function, and cognitive deficits in both humans and mouse models (Ahmed et al., 2022). Little is known about the causes of these pathologies, but patients with DS are suspected to be predisposed to developing AD late in life. García and Flores-Aguilar summarize data about glial cells in the context of DS-AD and inflammation.

Links for inflammation and glial cells could also exist in anorexia; food intake is reduced during acute and chronic inflammatory states in human and research models (Gautron and Layé, 2010). Reyes-Haro reviews features of physiological anorexia in research models in comparison with human pathological anorexia, emphasizing valid precautions when extrapolating results. Moreover, he discusses studies in murine models of anorexia in which glial cells putatively play central roles in classical hypothalamic mechanisms, as well as in systemic machineries including the prefrontal cortex. Specifically, the pro-inflammatory environment associated with microglia reactivity, the impact of astrocyte manipulation on food intake associated with purinergic gliotransmission, and the roles of Oligodendrocyte Precursor Cells (OPCs) mediating the anorexigenic action of leptin in mice, are presented.

Another pathology associated with glial cell dysfunction is alcohol exposure during pregnancy. Fetal Alcohol Syndrome (FAS) is a public health problem with a prevalence of 2–5% in the USA. FAS disturbs the structure and function of the brain, but the underlying mechanisms remain elusive (Holloway et al., 2023). Zheng et al., observed loss of the tubulin-binding cofactor B resulting in disorganized microtubules and shortening of astrocytic processes in a model of chronic alcohol exposure. Developmental pathological implications to consider include abnormal migration of neuronal precursors through aberrant radial glial processes and defective synaptic coverage by astrocytes.

Overall, articles in this topic cover diverse aspects of research on glial cells and serve as introductory information to several subfields of glial biology. Ideas presented encourage others to design studies to clarify the roles of physiological and pathological factors with potential use in therapeutic applications and engineering.

## Author contributions

DR-H, AL-J, and AR-C wrote the original draft and edited the final version of the manuscript. All authors contributed to the article and approved the submitted version.
